# Trends and Demographics of Hepatorenal Syndrome-Related Mortality in the U.S., 1999–2024: A CDC WONDER Analysis

**DOI:** 10.3390/diseases14030106

**Published:** 2026-03-12

**Authors:** Syed Faisal Ali, Julia Natche, Mahendrakumar Achlaram Chaudhari, Hassan Abbasi, Sammy Dawoud, Hany Dawoud, Amna Shoaib, Hersh Tilokani, Harleen Kaur Chela, Arsal Zafar

**Affiliations:** 1 Department of Medicine, Royal Devon and Exeter Hospital, Exeter EX2 5DW, UK; syedfaisal.ali@nhs.net; 2 Department of Medicine, University of Medicine and Health Sciences, Basseterre KN0101, Saint Kitts and Nevis; jnatche@umhs-sk.net; 3 Department of Medicine, Boston University, Boston, MA 02118, USA; mahencha@bu.edu; 4 Department of Medicine, Al Nafees Medical College, Islamabad 44000, Pakistan; hassanabbasi1239@gmail.com (H.A.); amnashoaib700@gmail.com (A.S.); 5 Department of Medicine, School of Medicine, International University of the Health Sciences, Basseterre KN0101, Saint Kitts and Nevis; sammyd@iuhs.edu (S.D.); hanyd@iuhs.edu (H.D.); 6 Department of Medicine, University of California, Los Angeles, CA 90095, USA; 7 Division of Gastroenterology and Hepatology, Charleston Area Medical Center, West Virginia University, Charleston, WV 25302, USA; 8 Department of Medicine, Islamabad Medical & Dental College, Islamabad 44000, Pakistan; arsal.22@imdcollege.edu.pk

**Keywords:** hepatorenal syndrome, mortality trends, CDC WONDER, health disparities, terlipressin

## Abstract

**Background:** Hepatorenal syndrome (HRS) is a severe complication of liver cirrhosis, marked by rapid renal function decline and poor prognosis. Although clinical predictors of HRS outcomes have been extensively studied, less is known about how demographic factors influence mortality patterns. **Methods:** This analysis utilized CDC WONDER data to assess U.S. mortality trends for hepatorenal syndrome (HRS) in adults aged ≥25 years from 1999 to 2024. We calculated crude mortality rates (CMR) and age-adjusted mortality rates (AAMR) per 100,000 and analyzed temporal trends using Joinpoint regression to determine the annual percentage change (APC) and average annual percentage change (AAPC). **Results:** From 1999 to 2024, 118,894 HRS-associated deaths were recorded. The overall AAMR decreased significantly from 2.43 in 1999 to 2.12 in 2024, with an AAPC of (AAPC −0.69% [95% CI: −0.90% to −0.51%]). Males consistently exhibited higher AAMRs than females (Males: 2.62 vs. Females: 1.63 in 2024). When stratified by race, the highest AAMR in 2024 was observed among non-Hispanic (NH) American Indian or Alaska Native populations (11.02), followed by Hispanic or Latino (2.58), NH White (2.23), NH Black or African American (1.30), and NH Asian or Pacific Islander populations (0.72). Regionally, the highest mortality was observed in the West, followed by the Midwest, South, and Northeast (2.88, 2.00, 1.92, and 1.53, respectively, in 2024). Rural areas (2.44) consistently exhibited higher AAMRs than urban areas (1.91) throughout the study period. **Conclusions:** HRS-related mortality has decreased modestly in the U.S over the last 26 years, yet significant inequities remain across population subgroups and regions. Mortality is disproportionately higher among males, NH American Indian or Alaska Native individuals, and residents of rural and western areas, highlighting the continued necessity for focused public health strategies.

## 1. Introduction

Hepatorenal syndrome (HRS) is a life-threatening complication of advanced cirrhosis and acute liver failure characterized by functional renal impairment driven by profound circulatory and neurohormonal dysregulation, leading to intense renal vasoconstriction and reduced glomerular filtration despite structurally normal kidneys [1,2,3]. Contemporary consensus frameworks have reframed the syndrome within the spectrum of acute kidney injury in cirrhosis (HRS-AKI), a rapidly progressive phenotype that frequently follows precipitating events such as infection, gastrointestinal bleeding, or large-volume paracentesis and may progress to multiorgan failure without timely intervention [4,5,6,7,8].

The clinical and health-system burden of HRS remains substantial. Prior U.S. studies have consistently associated HRS with high short-term mortality and significant resource utilization, including prolonged hospitalizations and greater costs compared with cirrhosis admissions without HRS [7,9,10,11,12]. In parallel, the broader epidemiology of chronic liver disease in the United States has evolved over the last two decades, with accelerating alcohol-associated liver disease (ALD) mortality particularly during and following the COVID-19 era and persistent disparities across age, sex, and race/ethnicity that may influence downstream complications such as HRS [13,14,15].

The treatment landscape has advanced, most notably with the approval of terlipressin by the U.S. Food and Drug Administration in 2022 as the first U.S.-approved pharmacologic therapy to improve kidney function in adults with HRS and rapid reduction in renal function [16]. Nevertheless, outcomes remain poor for many patients, especially those with advanced hepatic dysfunction or limited access to liver transplantation and specialized liver care [16,17]. These evolving clinical and epidemiologic contexts underscore the importance of defining long-term mortality patterns and identifying population subgroups at disproportionate risk.

Despite condition-specific studies examining HRS-AKI and cirrhosis-related AKI, long-horizon, population-level evaluations of HRS-related mortality that quantify temporal inflection points and disparities by sex, race/ethnicity, geography, and urban–rural residence remain limited [9,10,18]. Therefore, using the CDC WONDER Multiple Cause of Death database, we examined the U.S. HRS-related mortality trends from 1999 through 2024 and assessed differences by sex, age group, race/ethnicity, census region, state, and urbanization level. We hypothesized that (i) HRS-related mortality rates have changed nonlinearly over time, with identifiable periods of acceleration and decline, and (ii) these trends exhibit persistent and clinically meaningful disparities across demographic and geographic subgroups.

## 2. Methods

### 2.1. Study Design and Database

This retrospective, population-based mortality analysis estimated U.S. deaths associated with hepatorenal syndrome (HRS) from 1999 through 2024 using the Centers for Disease Control and Prevention Wide-ranging Online Data for Epidemiologic Research (CDC WONDER) Multiple Cause of Death (MCD) platform. The study period was selected because national ICD-10–coded MCD data are available beginning in 1999, and the analysis was extended through the most recently available year at the time of extraction (2024). Because CDC WONDER includes both final MCD files (1999–2020 and 2018–2023 single-race) and a provisional MCD series for deaths occurring in 2018 through the most recent available week/year, results for the most recent year were interpreted with the understanding that provisional counts may be updated as records are finalized.

Deaths were eligible if HRS (ICD-10: K76.7) was listed as either the underlying cause of death or a contributing (multiple) cause on the death certificate among adults aged ≥25 years. This case-identification approach is consistent with prior CDC-WONDER-based analyses of HRS mortality. For reproducibility, all CDC WONDER requests for overall and subgroup analyses were generated using a consistent set of filters (age ≥ 25 years; ICD-10 K76.7 as underlying or multiple cause), and outputs were exported contemporaneously to minimize the impact of subsequent database updates. Additionally, we adhered to the Strengthening the Reporting of Observational Studies in Epidemiology (STROBE) reporting guidance. Institutional review board approval was not required because this study used de-identified, publicly available mortality data.

### 2.2. Demographic and Geographical Study Groups

Extracted variables included sex, race/ethnicity, age group, urbanization level, and geographic location (state and U.S. Census region). Sex was classified as male or female. Race/ethnicity was categorized as non-Hispanic (NH) White, NH Black or African American, NH American Indian or Alaska Native (AI/AN), NH Asian/Pacific Islander, and Hispanic/Latino, consistent with CDC WONDER race/ethnicity reporting formats used across the MCD series; where reporting structures differed between bridged-race (1999–2020) and single-race (2018–2023 and provisional), categories were harmonized to these analytic groupings to enable longitudinal comparisons.

Participants were stratified into three age groups: 25–44 years (younger adults), 45–64 years (middle-aged adults), and ≥65 years (older adults). Urbanization status was defined using the National Center for Health Statistics (NCHS) Urban–Rural Classification Scheme for Counties (URCS). Counties were grouped as metropolitan (large metropolitan and medium/small metropolitan) versus nonmetropolitan (micropolitan and noncore), consistent with standard URCS-based approaches and CDC documentation. Geographic analyses used the four U.S. Census regions (Northeast, Midwest, South, West) and state of residence.

### 2.3. Statistical Analysis

To quantify mortality, we calculated crude mortality rates (CMR) and age-adjusted mortality rates (AAMR) per 100,000 population. The CMR was computed as deaths divided by the corresponding U.S. population estimate. AAMRs were calculated using direct standardization with the year 2000 U.S. standard population, applied consistently across all demographic and geographic subgroup analyses. Temporal trends in AAMR were evaluated using the Joinpoint Regression Program (Joinpoint; version 5.4.0). The Joinpoint software fits the simplest model supported by the data, beginning with the minimum number of joinpoints and testing whether additional joinpoints significantly improve model fit up to a user-specified maximum, using permutation testing. For each identified segment, we report the annual percent change (APC) with 95% confidence intervals (CIs). The overall trend is summarized by the average annual percent change (AAPC), representing the weighted average of segment-specific APCs. Statistical significance of APC/AAPC estimates was assessed using two-sided tests with α = 0.05.

Handling of suppressed/missing data: CDC WONDER suppresses cells for small counts in certain stratified outputs to protect confidentiality. In subgroup analyses where suppression occurred, affected strata-years were treated as missing and were not imputed; where feasible, results were derived from higher-level aggregations available without suppression (e.g., region-level rather than county-level). Trends for small subgroups were interpreted cautiously given the potential instability of rate estimates when based on low event counts.

Sensitivity/robustness assessments: To address potential artifacts from reporting changes, provisional updates, and extreme fluctuations, we assessed the stability of main trend inferences by comparing results using final-data windows (where available) versus the full series inclusive of the most recent year, and by verifying that key inflection periods persisted when restricting analyses to intervals less influenced by anomalous spikes.

## 3. Results

All rates are reported per 100,000 population. Analyses were based on CDC WONDER Multiple Cause of Death data (1999–2024); the most recent year reflects the latest available data at the time of extraction and may be subject to subsequent update. Although CDC WONDER may suppress small cells in some stratified outputs, no suppression was encountered in the datasets used for this analysis; accordingly, no strata-years were missing and no imputation was performed.

### 3.1. Overall

**Figure 1:**
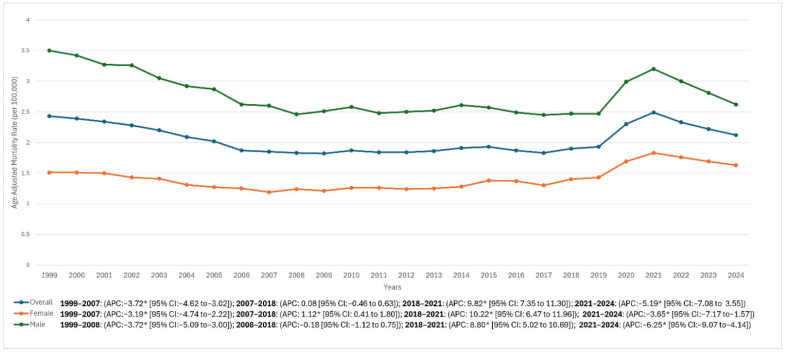
HRS-associated AAMRs per 100,000 stratified by sex in the United States from 1999 to 2024. Age-adjusted mortality rates (AAMR) per 100,000 for hepatorenal syndrome (HRS) among U.S. adults aged ≥25 years, 1999–2024, stratified by sex. Rates were calculated using CDC WONDER Multiple Cause of Death data with ICD-10 code K76.7 listed as an underlying or contributing cause of death and were age-adjusted to the 2000 U.S. standard population. Joinpoint regression identified significant changes in temporal trends; segment-specific annual percent change (APC) and 95% confidence intervals (CI) are shown in the figure/inset (overall, male, female). * Indicates APC is statistically significant (*p* < 0.05). Abbreviations: AAMR, age-adjusted mortality rate; APC, annual percent change; CI, confidence interval; HRS, hepatorenal syndrome.

Overall, there was a modest decline in AAMR from 1999 to 2024, with rates decreasing from 2.43 in 1999 to 2.12 in 2024 (AAPC −0.69% [95% CI: −0.90% to −0.51%]). The trend analysis revealed an initial marked decline between 1999 and 2007 (APC −3.72% [95% CI: −4.62% to −3.02%]), followed by a prolonged period of stability from 2007 to 2018 (APC 0.08% [95% CI: −0.46% to 0.63%]). Subsequently, a sharp increase was observed between 2018 and 2021 (APC 9.82% [95% CI: 7.35% to 11.30%]), after which rates declined significantly from 2021 to 2024 (APC −5.19% [95% CI: −7.08% to −3.55%]) (Figure 1, Table 1, and Supplementary Table S1).

### 3.2. Sex

Among males, the AAMR declined sharply from 3.50 in 1999 to 2.60 in 2008 (APC −3.72% [95% CI: −5.09% to −3.00%]). The subsequent period from 2008 to 2018 demonstrated relative stability, with rates fluctuating around 2.47 in 2018 and a non-significant APC of −0.18% (95% CI: −1.12% to 0.75%). A pronounced surge followed between 2018 and 2021, as AAMR rose to 3.20 (APC 8.80% [95% CI: 5.02% to 10.69%]), before reversing with a marked decline to 2.62 in 2024 (APC −6.25% [95% CI: −9.07% to −4.14%]). Over the entire study period (1999–2024), the male AAMR exhibited an overall significant downward trend (AAPC −1.19% [95% CI: −1.48% to −0.97%]).

Among females, AAMR decreased from 1.51 in 1999 to 1.19 in 2007 (APC −3.19% [95% CI: −4.74% to −2.22%]), followed by a gradual increase reaching 1.40 in 2018 (APC 1.12% [95% CI: 0.41% to 1.80%]). A sharp escalation occurred through 2021, peaking at 1.83 (APC 10.22% [95% CI: 6.47% to 11.96%]), after which rates declined to 1.63 in 2024 (APC −3.65% [95% CI: −7.17% to −1.57%]). Across the full 1999–2024 interval, the overall trend among females was not statistically significant (AAPC 0.18% [95% CI: −0.13% to 0.43%]) (Figure 1, Table 1, and Supplementary Table S1).

### 3.3. Race

Among NH Whites, AAMR declined from 2.20 in 1999 to 1.79 in 2012 (APC −3.25% [95% CI: −5.21 to −2.31]), then stabilized around 1.92 in 2018 (APC 0.63% [95% CI: −0.12 to 1.41]), before a spike to 2.57 in 2021 (APC 10.85% [95% CI: 6.18–12.69]); this was followed by a decline to 2.23 in 2024 (APC −4.29% [95% CI: −7.54 to −2.14]), with an overall AAPC of −0.07% (95% CI: −0.40 to 0.18).

Among Hispanics/Latinos, AAMR decreased from 4.35 in 1999 to 2.53 in 2018 (APC −5.54% [95% CI: −7.55 to −4.16]), followed by a modest increase through 2021 (AAMR 2.85) and a decline to 2.58 in 2024 (segment APC 2008–2024: 0.27% [95% CI: −0.24 to 0.91]; overall AAPC −1.86% [95% CI: −2.20 to −1.49]).

Among NH Black/African American individuals, AAMR decreased from 2.82 in 1999 to 1.84 in 2007 (APC −5.95% [95% CI: −10.77 to −3.97]) and continued to decline to 1.30 in 2024 (APC 2007–2024: −1.38% [95% CI: −2.12 to −0.16]; overall AAPC −2.87% [95% CI: −3.38 to −2.35]).

Among NH Asian/Pacific Islander individuals, AAMR declined from 1.93 in 1999 to 0.72 in 2024, with an overall AAPC of −3.27% (95% CI: −3.97 to −2.53).

**Figure 2:**
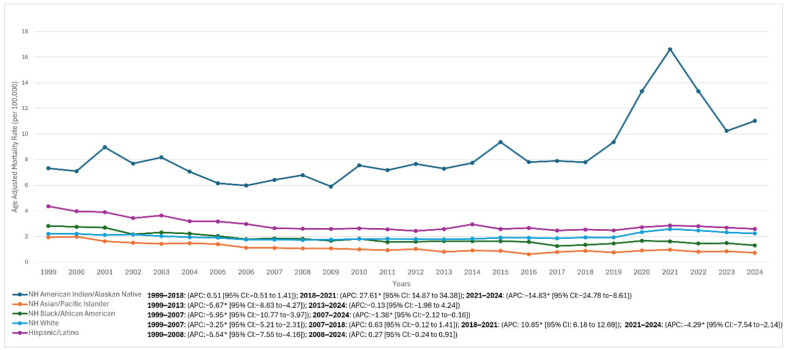
HRS-associated AAMRs per 100,000 stratified by race in the United States from 1999 to 2024. Age-adjusted mortality rates (AAMR) per 100,000 for hepatorenal syndrome (HRS) among U.S. adults aged ≥25 years, 1999–2024, stratified by race/ethnicity. Deaths were identified in CDC WONDER Multiple Cause of Death data using ICD-10 code K76.7 listed as an underlying or contributing cause of death; rates were age-adjusted to the 2000 U.S. standard population. Joinpoint regression segment-specific annual percent change (APC) and 95% CIs are provided in the figure/inset for each group. * Indicates APC is statistically significant (*p* < 0.05). Definitions: NH = non-Hispanic. Interpretation of year-to-year fluctuations should consider smaller subgroup counts and CDC WONDER suppression rules that may affect stability of estimates.

Among NH American Indian/Alaska Native individuals, rates were relatively stable from 7.32 in 1999 to 7.79 in 2018 (APC 0.51% [95% CI: −0.51 to 1.41]) before a sharp rise to 16.61 in 2021 (APC 27.61% [95% CI: 14.87–34.38]) and a subsequent fall to 11.02 in 2024 (APC −14.83% [95% CI: −24.78 to −8.61]); the overall AAPC for this group was 1.40% (95% CI: 0.61–2.15). Given smaller absolute counts in some strata, subgroup trend estimates were interpreted with caution where suppression or instability may occur. (Figure 2, Table 1, and Supplementary Table S2).

### 3.4. Region

Adults in different U.S. Census regions consistently exhibited varying AAMR throughout the study period. In the Northeast, AAMR declined from 2.38 in 1999 to 1.53 in 2024 (AAPC −1.77% [95% CI: −2.06 to −1.55]). In the Midwest, AAMR changed from 2.08 in 1999 to 2.00 in 2024 (AAPC −0.02% [95% CI: −0.32 to 0.25]). In the South, AAMR decreased from 2.40 in 1999 to 1.92 in 2024 (AAPC −0.96% [95% CI: −1.28 to −0.69]). In the West, AAMR decreased slightly from 2.92 in 1999 to 2.88 in 2024 (AAPC −0.06% [95% CI: −0.37 to 0.28]) (Table 1, Supplementary Figure S1, and Supplementary Table S3).

### 3.5. Urbanization

Urbanization-stratified Joinpoint analyses were available through 2020 in the current supplementary outputs; therefore, urbanization results are reported for 1999–2020 to maintain internal consistency across the urban/rural subgroup estimates presented (Supplementary Figure S2 and Supplementary Table S4).

In metropolitan areas, the AAMR declined from 2.38 in 1999 to 1.76 in 2007 (APC −4.09% [95% CI: −5.63% to −3.23%]), followed by minimal change to 1.78 in 2018 (APC 0.02% [95% CI: −0.94% to 0.74%]). This was followed by an increase to 2.13 in 2020 (APC 8.45% [95% CI: 2.95% to 11.25%]). Over 1999–2020, metropolitan areas demonstrated an overall decline (AAPC −0.80% [95% CI: −1.16% to −0.53%]).

In non-metropolitan areas, AAMR declined from 2.60 in 1999 to 2.16 in 2009 (APC −1.85% [95% CI: −8.15% to −0.19%]), followed by an upward trend reaching 3.18 in 2020 (APC 2.35% [95% CI: 0.92% to 7.56%]). Across 1999–2020, the overall trend in non-metropolitan areas did not reach statistical significance (AAPC 0.33% [95% CI: −0.42% to 1.10%]) (Supplementary Figure S2 and Supplementary Table S4).

### 3.6. States

**Figure 3:**
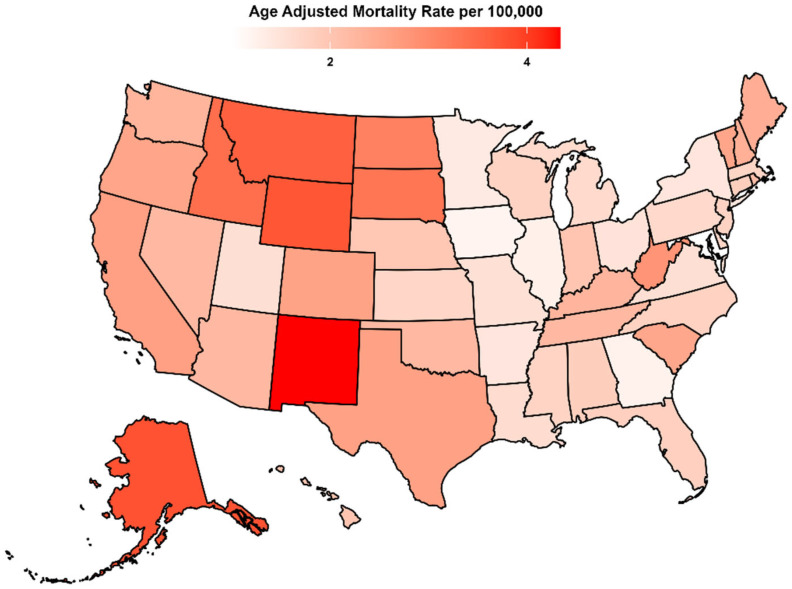
HRS-associated AAMRs per 100,000 stratified by state in the United States from 1999 to 2024. Abbreviations as in Figure 1. State-level age-adjusted mortality rate (AAMR) per 100,000 for hepatorenal syndrome (HRS) among U.S. adults aged ≥25 years, aggregated across 1999–2024. Rates were derived from CDC WONDER Multiple Cause of Death data using ICD-10 code K76.7 and age-adjusted to the 2000 U.S. standard population. Darker shading indicates higher mortality.

State-level AAMR varied widely, ranging from 1.26 in Maryland (95% CI: 1.19 to 1.34) to 4.40 in New Mexico (95% CI: 4.16 to 4.65). For clarity, states with the lowest AAMRs (lowest decile) and the highest AAMRs (highest decile) are provided in Supplementary Table S5, and the geographic distribution is shown in Figure 3 (Figure 3, Table 1, and Supplementary Table S5).

### 3.7. Age

Because age adjustment is not applied within age strata, age-group analyses are reported as crude (age-specific) mortality rates (CMR) for each age band.

Among younger adults aged 25–44, CMR declined from 0.68 in 1999 to 0.46 in 2006 (APC −5.84% [95% CI: −10.97% to −3.65%]) before stabilizing at 0.44 in 2017 (APC 0.04% [95% CI: −1.25% to 2.32%]). Rates then increased to 0.95 in 2021 (APC 19.64% [95% CI: 14.05% to 28.37%]) before decreasing to 0.81 in 2024 (APC −6.27% [95% CI: −11.81% to −1.16%]). Across 1999–2024, the AAPC for this cohort was 0.43% (95% CI: −0.15% to 0.95%).

Among middle-aged adults (45–64), CMR decreased from 3.19 in 1999 to 2.68 in 2007 (APC −2.27% [95% CI: −3.44% to −1.50%]), increased to 3.02 in 2015 (APC 1.12% [95% CI: 0.56% to 3.05%]), declined to 2.66 in 2018 (APC −4.21% [95% CI: −5.90% to −2.18%]), increased to 3.63 in 2021 (APC 11.70% [95% CI: 9.55% to 13.81%]), and then decreased to 2.74 in 2024 (APC −8.84% [95% CI: −10.86% to −7.07%]).

Among older adults (≥65), CMR decreased from 5.22 in 1999 to 3.37 in 2009 (APC −4.70% [95% CI: −5.94% to −3.81%]) before increasing to 4.19 in 2024 (APC 1.82% [95% CI: 1.34% to 2.40%]). The corresponding full-period AAPCs were −0.67% (95% CI: −0.90% to −0.48%) for middle-aged adults and −0.84% (95% CI: −1.09% to −0.56%) for older adults (Supplementary Figure S3 and Supplementary Table S6).

## 4. Discussion

This study provides a comprehensive population-based evaluation of HRS-related mortality in the United States using CDC WONDER Multiple Cause of Death data from 1999 to 2024. Overall mortality rates declined modestly over the study period, with an early reduction, a prolonged interval of relative stability, a sharp surge between 2018 and 2021, and a subsequent decline. Men exhibited higher mortality than women, and non-Hispanic American Indian/Alaska Native (NH AI/AN) populations had the greatest burden. Mortality rates were higher in non-metropolitan areas than in metropolitan areas (urbanization outputs available through 2020), while the Western region consistently exhibited the highest mortality. There was substantial geographic variation, with some states demonstrating more than threefold higher mortality rates than others.

The observed temporal inflection points particularly the abrupt rise during 2018–2021 and decline thereafter should be interpreted cautiously. Such changes may reflect true epidemiologic shifts, but they can also be influenced by changes in underlying liver disease incidence, disruption in healthcare access during the COVID-19 era, evolving cause-of-death certification practices, and provisional-to-final file updates in more recent years. Accordingly, we emphasize these patterns as population-level trend signals rather than definitive evidence of causal mechanisms.

Our findings demonstrate a clear sex-based disparity in long-term HRS mortality trends, with men bearing a persistently higher mortality burden and demonstrating a significant decline over time, whereas rates among women remained largely unchanged. This pattern has not been consistently characterized in prior population-based reports, as many trend analyses have not focused on sex-stratified mortality trajectories [9,19]. The higher mortality burden among men is consistent with the higher population burden of alcohol-associated liver disease (ALD), a major upstream driver of cirrhosis decompensation and complications including HRS [20]. In addition, prior nationwide data suggest sex differences in outcomes following transjugular intrahepatic portosystemic shunt (TIPS) among patients hospitalized with HRS, though the underlying mechanisms remain incompletely defined [21]. Importantly, our ecological design cannot distinguish whether these sex differences reflect variation in disease incidence, survival after HRS onset, treatment access (including transplant referral), or differential reporting on death certificates [22,23,24]. Future studies linking clinical cohorts to outcomes are needed to clarify these pathways.

The results also highlight substantial racial and ethnic disparities in HRS-related mortality. Mortality rates varied markedly across groups, with the highest AAMRs observed among NH AI/AN population and representing the only group with a significant long-term increase. While this disparity is concerning and aligns with broader inequities in chronic liver disease outcomes [25], interpretation should account for potential instability of rate estimates in smaller subgroups and for CDC WONDER suppression in certain stratified outputs. Structural factors plausibly contributing to these differences include differential access to specialty hepatology care, transplantation, insurance coverage, and higher burdens of ALD and viral hepatitis in some communities; however, because CDC WONDER does not contain individual-level socioeconomic or clinical variables, these explanations should be considered hypothesis-generating rather than causal conclusions [25,26,27,28,29,30]. The improvement observed in NH Black populations may reflect advances in prevention and management, though persistent barriers to timely advanced therapies remain documented in the broader liver disease literature [28,29,30].

Urbanization-stratified trends suggest that HRS mortality in non-metropolitan areas surpassed metropolitan regions after 2009 (reported through 2020 in the current supplementary outputs). This pattern is consistent with broader rural health inequities, including higher burdens of chronic disease and reduced access to specialty services [31,32,33]. Notably, national surveillance has documented increasing alcohol-induced death rates in both urban and rural counties over the last two decades, with higher rates in rural areas by 2020, which may contribute to rural liver disease severity and downstream complications such as HRS [34,35]. Nevertheless, these interpretations remain indirect; our data cannot evaluate individual alcohol exposure, comorbidities, or care pathways, underscoring the need for future work integrating mortality patterns with healthcare access and treatment utilization measures.

Age-stratified analyses revealed divergent trajectories in HRS-related mortality. Among younger adults, the pronounced rise since the late 2010s may reflect increasing burdens of ALD and substance-associated liver injury, which intensified during the COVID-19 era alongside documented increases in population-level alcohol use and alcohol-related harms [36,37,38]. In contrast, older adults experienced a sustained long-term decline beginning in 1999, potentially consistent with incremental improvements in prevention, infection control, and cirrhosis management [39]. However, mortality in older adults has risen gradually since approximately 2009, which may relate to population aging and increasing multimorbidity and frailty, factors that can worsen outcomes in decompensated cirrhosis and renal dysfunction [39,40,41]. Because our analysis reports mortality rates rather than survival among clinically diagnosed HRS cases, these age patterns likely reflect the combined effects of changes in HRS incidence, competing risks, and survival after onset, none of which can be disentangled within CDC WONDER.

From a clinical and public health perspective, the growing divergence across age, sex, and geography emphasizes that progress in HRS management has not translated equally across the population. Interventions to reduce mortality should focus on prevention and early recognition of cirrhosis decompensation, rapid initiation of albumin and guideline-concordant vasoconstrictor therapy, and streamlined referral pathways to centers capable of advanced liver care, including transplant evaluation. The approval of terlipressin in 2022 provides an additional therapeutic option for HRS-AKI, but equitable access and appropriate utilization depend on clinician awareness, infrastructure to monitor adverse effects, and timely diagnosis [15]. Strengthening rural health infrastructure (including tele-hepatology), integrating alcohol-use prevention and treatment services, and expanding hepatitis screening and linkage-to-care programs may help address upstream drivers of HRS risk and mitigate observed disparities [31,32,33,34,35]. Collectively, sustained reductions in HRS mortality will likely require both therapeutic innovation and systems-level efforts to improve equitable access to specialty liver care in the United States.

## 5. Study Limitations

This study has several limitations. First, reliance on death certificate data from CDC WONDER may introduce miscoding or misclassification of underlying and contributing causes of death; certification errors and ICD-10 coding practices can meaningfully affect cause-specific mortality statistics. Second, the database is limited to mortality information and does not include individual-level clinical variables (e.g., cirrhosis severity, precipitating events, laboratory measures), treatment details (including vasoconstrictor use, renal replacement therapy, or transplant evaluation), or socioeconomic measures, restricting adjustment for confounding and precluding assessment of survival after HRS onset.

Third, ICD-10 code K76.7 may not fully capture all HRS deaths, as HRS can be recorded under broader renal failure or cirrhosis-related codes and depends on clinician recognition and documentation at the time of death certification; changes in coding guidance and reporting practice over time may influence comparability. Fourth, CDC WONDER applies confidentiality suppression rules for small counts in certain stratified outputs, and small subgroup estimates may be unstable; therefore, trend estimates for smaller strata (e.g., certain race/ethnicity-by-region cells) should be interpreted cautiously.

Fifth, the most recent years may include frequently updated/provisional files, and reported rates particularly for the latest year may change as records are finalized or revised. Sixth, although we used consistent age-adjustment methods (year 2000 U.S. standard), differences in race/ethnicity reporting frameworks across CDC WONDER series (e.g., bridged-race vs single-race) and potential changes in classification practices over time may affect long-term subgroup comparisons despite harmonization efforts.

Finally, because this is an observational, population-level mortality analysis, causal relationships cannot be inferred, and findings may not generalize beyond U.S. residents.

## 6. Conclusions

HRS-related mortality in the U.S. declined modestly from 1999–2024, with an early drop, long stability, a spike in 2018–2021, and a subsequent decline. Mortality remained higher in men than women, was highest among non-Hispanic American Indian/Alaska Native individuals (the only group with a sustained long-term increase), and showed persistent geographic disparities especially higher rates in the West and in non-metropolitan areas (reported through 2020 in Supplementary Analyses). A recent rise among younger adults suggests an emerging high-risk group, reinforcing the need for prevention of upstream liver disease (ALD/viral hepatitis), earlier recognition, and more equitable access to specialized liver care and advanced therapies nationwide.

## Figures and Tables

**Table 1:** Frequency and Age-Adjusted Mortality Rates per 100,000 in HRS, Stratified by Sex, Race, and Census Region.

	Deaths	Population	AAMR 1999 (95% CI)	AAMR 2024 (95% CI)	AAPC (95% CI)
**Overall**	118,894	5.39 × 10^9^	2.43 (2.36 to 2.51)	2.12 (2.06 to 2.18)	−0.69 * (−0.9 to −0.51)
**Sex**					
**Men**	75,646	2.60 × 10^9^	3.50 (3.37–3.63)	2.62 (2.52–2.71)	−1.19 * (−1.48 to −0.97)
**Women**	43,248	2.79 × 10^9^	1.51 (1.43–1.58)	1.63 (1.56–1.70)	0.18 (−0.13 to 0.43)
**NH race**					
**NH American Indian/Alaskan Native**	3347	3.95 × 10^7^	7.32 (5.78–9.15)	11.02 (9.33–12.70)	1.40 * (0.61 to 2.15)
**NH White**	84,509	3.68 × 10^9^	2.20 (2.13–2.28)	2.23 (2.15–2.30)	−0.07 (−0.4 to 0.18)
**NH Black/African American**	11,070	6.30 × 10^8^	2.82 (2.57–3.07)	1.30 (1.17–1.44)	−2.87 * (−3.38 to −2.35)
**NH Asian/Pacific Islander**	2634	2.97 × 10^8^	1.93 (1.54–2.38)	0.72 (0.58–0.86)	−3.27 * (−3.97 to −2.53)
**Hispanic race**	16,863	7.40 × 10^8^	4.35 (3.95–4.75)	2.58 (2.41–2.75)	−1.86 * (−2.2 to −1.49)
**Census Region**					
**Northeast**	19,077	9.88 × 10^8^	2.38 (2.22–2.54)	1.53 (1.41–1.64)	−1.77 * (−2.06 to −1.55)
**Midwest**	22,359	1.16 × 10^9^	2.08 (1.94–2.22)	2.00 (1.88–2.12)	−0.02 (−0.32 to 0.25)
**South**	43,318	2.00 × 10^9^	2.40 (2.28–2.52)	1.92 (1.83–2.01)	−0.96 * (−1.28 to −0.69)
**West**	34,140	1.24 × 10^9^	2.92 (2.75–3.10)	2.88 (2.74–3.02)	−0.06 (−0.37 to 0.28)

* Indicates statistical significance (*p* < 0.05). AAMR = age-adjusted mortality rate; AAPC = average annual percent change; HRS = hepatorenal syndrome; NH = non-Hispanic.

## Data Availability

The data analyzed in this study were obtained from the CDC WONDER database, which provides access to a wide range of public health datasets. The mortality data used in this analysis are publicly available and can be accessed through the CDC WONDER website at https://wonder.cdc.gov/ (accessed on 5 September 2025). Any additional datasets generated or analyzed during this study are available from the corresponding author upon reasonable request.

## Supplementary Materials

The following supporting information can be downloaded at: https://www.mdpi.com/article/10.3390/diseases14030106/s1, Figure S1: HRS-associated AAMRs per 100,000 stratified by census region in the United States from 1999 to 2024; Figure S2: HRS-associated AAMRs per 100,000 stratified by urbanization in the United States from 1999 to 2020; Figure S3: HRS-associated AAMRs per 100,000 stratified by age groups in the United States from 1999 to 2024; Table S1: Overall and sex-stratified HRS-related AAMR per 100,000 in the United States from 1999 to 2024; Table S2: HRS-related AAMR per 100,000 stratified by Race in the United States from 1999 to 2024; Table S3: HRS-related AAMR per 100,000 stratified by Census Region in the United States from 1999 to 2024; Table S4: HRS-related AAMR per 100,000 stratified by Urban-Rural classification in the United States from 1999 to 2020; Table S5: HRS-related AAMR per 100,000 stratified by State in the United States from 1999 to 2020; Table S6: HRS-related CMR stratified by age groups in the United States from 1999 to 2024.
