# Supplementary material for: Trends and Demographics of Hepatorenal Syndrome-Related Mortality in the U.S., 1999–2024: A CDC WONDER Analysis
Source: Diseases. 2026 Mar 12;14(3):106. doi: 10.3390/diseases14030106 (PMC13024754; doi:10.3390/diseases14030106)
Supplement: Supplementary file 1 [file diseases-14-00106-s001.zip › diseases-4123230-supplementary.pdf]

Supplemental Table S1. Overall and sex-stratified HRS-related AAMR per 100,000 in the United States from 1999 to 2024.

	Age-Adjusted Mortality Rate (95% CI)		
**Year**	**Men**	**Women**	**Overall**
**1999**	3.50 (3.37-3.63)	1.51 (1.43-1.58)	2.43 (2.36-2.51)
**2000**	3.42 (3.29-3.55)	1.51 (1.43-1.59)	2.39 (2.32-2.46)
**2001**	3.27 (3.15-3.40)	1.50 (1.43-1.58)	2.34 (2.27-2.41)
**2002**	3.26 (3.14-3.38)	1.43 (1.36-1.51)	2.28 (2.21-2.35)
**2003**	3.05 (2.93-3.17)	1.41 (1.34-1.48)	2.20 (2.14-2.27)
**2004**	2.92 (2.81-3.03)	1.31 (1.24-1.38)	2.09 (2.02-2.15)
**2005**	2.87 (2.76-2.99)	1.27 (1.20-1.34)	2.02 (1.95-2.08)
**2006**	2.62 (2.51-2.72)	1.25 (1.18-1.31)	1.87 (1.81-1.93)
**2007**	2.60 (2.50-2.70)	1.19 (1.12-1.25)	1.85 (1.79-1.91)
**2008**	2.46 (2.36-2.55)	1.24 (1.18-1.31)	1.83 (1.77-1.89)
**2009**	2.51 (2.41-2.60)	1.21 (1.15-1.28)	1.82 (1.77-1.88)
**2010**	2.58 (2.48-2.68)	1.26 (1.20-1.33)	1.87 (1.81-1.93)
**2011**	2.48 (2.39-2.58)	1.26 (1.20-1.33)	1.84 (1.78-1.90)
**2012**	2.50 (2.40-2.59)	1.24 (1.17-1.30)	1.84 (1.78-1.89)
**2013**	2.52 (2.43-2.62)	1.25 (1.18-1.31)	1.86 (1.80-1.91)
**2014**	2.61 (2.51-2.70)	1.28 (1.22-1.34)	1.91 (1.85-1.96)
**2015**	2.57 (2.48-2.67)	1.38 (1.32-1.45)	1.93 (1.87-1.99)
**2016**	2.49 (2.40-2.58)	1.37 (1.30-1.43)	1.87 (1.81-1.92)
**2017**	2.45 (2.36-2.54)	1.30 (1.23-1.36)	1.83 (1.78-1.88)
**2018**	2.47 (2.37-2.56)	1.40 (1.34-1.47)	1.90 (1.84-1.95)
**2019**	2.47 (2.38-2.56)	1.43 (1.37-1.50)	1.93 (1.88-1.99)
**2020**	2.99 (2.89-3.09)	1.69 (1.61-1.76)	2.30 (2.24-2.36)
**2021**	3.20 (3.10-3.31)	1.83 (1.76-1.91)	2.49 (2.43-2.56)

**2022**	3.00 (2.90-3.10)	1.76 (1.68-1.83)	2.33 (2.27-2.39)
**2023**	2.81 (2.72-2.91)	1.69 (1.62-1.76)	2.22 (2.16-2.28)
**2024**	2.62 (2.52-2.71)	1.63 (1.56-1.70)	2.12 (2.06-2.18)

Supplemental Table S2. HRS-related AAMR per 100,000 stratified by Race in the United States from 1999 to 2024.

	**Age-Adjusted Mortality Rate (95% CI)**				
**Year**	**NH American Indian/Alaska Native**	**NH Asian/Pacific Islander**	**NH Black/African American**	**NH White**	**Hispanic/Latino**
**1999**	7.32 (5.78-9.15)	1.93 (1.54-2.38)	2.82 (2.57-3.07)	2.20 (2.13-2.28)	4.35 (3.95-4.75)
**2000**	7.09 (5.60-8.86)	1.98 (1.59-2.37)	2.74 (2.49-2.99)	2.20 (2.12-2.27)	3.96 (3.59-4.34)
**2001**	8.96 (7.15-10.77)	1.62 (1.30-2.00)	2.69 (2.44-2.93)	2.11 (2.03-2.18)	3.89 (3.54-4.24)
**2002**	7.68 (6.16-9.45)	1.50 (1.20-1.85)	2.16 (1.94-2.37)	2.15 (2.08-2.23)	3.43 (3.11-3.75)
**2003**	8.17 (6.55-9.80)	1.42 (1.14-1.74)	2.31 (2.09-2.54)	2.02 (1.94-2.09)	3.63 (3.30-3.95)
**2004**	7.06 (5.63-8.74)	1.47 (1.17-1.82)	2.22 (2.01-2.43)	1.94 (1.87-2.01)	3.18 (2.89-3.47)
**2005**	6.15 (4.91-7.61)	1.40 (1.13-1.72)	2.03 (1.83-2.23)	1.90 (1.83-1.97)	3.16 (2.87-3.44)
**2006**	5.97 (4.67-7.52)	1.11 (0.88-1.39)	1.78 (1.60-1.96)	1.75 (1.68-1.82)	2.97 (2.70-3.24)
**2007**	6.41 (5.14-7.91)	1.10 (0.88-1.37)	1.84 (1.65-2.02)	1.74 (1.68-1.81)	2.64 (2.40-2.89)
**2008**	6.78 (5.40-8.16)	1.06 (0.85-1.30)	1.81 (1.63-1.99)	1.72 (1.66-1.79)	2.60 (2.36-2.84)
**2009**	5.89 (4.67-7.33)	1.06 (0.85-1.32)	1.66 (1.49-1.83)	1.75 (1.68-1.81)	2.58 (2.34-2.81)
**2010**	7.54 (6.09-8.99)	0.99 (0.79-1.24)	1.81 (1.63-1.98)	1.80 (1.74-1.87)	2.62 (2.39-2.85)
**2011**	7.17 (5.78-8.57)	0.92 (0.73-1.14)	1.56 (1.40-1.72)	1.81 (1.75-1.88)	2.56 (2.33-2.78)
**2012**	7.66 (6.22-9.10)	1.02 (0.82-1.22)	1.59 (1.43-1.75)	1.79 (1.73-1.86)	2.43 (2.22-2.64)
**2013**	7.29 (5.97-8.62)	0.80 (0.64-1.00)	1.63 (1.47-1.79)	1.77 (1.71-1.84)	2.57 (2.35-2.78)
**2014**	7.74 (6.34-9.14)	0.90 (0.72-1.07)	1.63 (1.47-1.79)	1.81 (1.74-1.87)	2.94 (2.72-3.16)
**2015**	9.37 (7.85-10.89)	0.87 (0.70-1.04)	1.63 (1.47-1.78)	1.90 (1.84-1.97)	2.57 (2.36-2.77)
**2016**	7.80 (6.43-9.17)	0.61 (0.48-0.77)	1.57 (1.42-1.72)	1.89 (1.83-1.96)	2.66 (2.46-2.86)
**2017**	7.89 (6.51-9.27)	0.78 (0.63-0.94)	1.25 (1.12-1.39)	1.85 (1.78-1.91)	2.46 (2.28-2.65)

**2018**	7.79 (6.42-9.15)	0.88 (0.72-1.04)	1.34 (1.20-1.48)	1.92 (1.85-1.99)	2.53 (2.34-2.72)
**2019**	9.36 (7.88-10.83)	0.75 (0.61-0.90)	1.45 (1.31-1.60)	1.92 (1.85-1.99)	2.47 (2.29-2.66)
**2020**	13.33 (11.57-15.08)	0.90 (0.75-1.06)	1.66 (1.51-1.81)	2.34 (2.26-2.41)	2.72 (2.54-2.90)
**2021**	16.61 (14.53-18.69)	0.96 (0.80-1.13)	1.61 (1.46-1.76)	2.57 (2.49-2.65)	2.85 (2.67-3.04)
**2022**	13.34 (11.48-15.20)	0.80 (0.66-0.95)	1.44 (1.30-1.58)	2.46 (2.38-2.53)	2.80 (2.62-2.98)
**2023**	10.23 (8.63-11.83)	0.84 (0.69-0.98)	1.48 (1.34-1.63)	2.32 (2.24-2.39)	2.68 (2.50-2.85)
**2024**	11.02 (9.33-12.70)	0.72 (0.58-0.86)	1.30 (1.17-1.44)	2.23 (2.15-2.30)	2.58 (2.41-2.75)

Supplemental Table S3. HRS-related AAMR per 100,000 stratified by Census Region in the United States from 1999 to 2024.

	**Age Adjusted Mortality Rate (95% CI)**			
**Year**	**Northeast**	**Midwest**	**South**	**West**
**1999**	2.38 (2.22-2.54)	2.08 (1.94-2.22)	2.40 (2.28-2.52)	2.92 (2.75-3.10)
**2000**	2.18 (2.03-2.33)	1.96 (1.83-2.10)	2.41 (2.29-2.54)	3.01 (2.84-3.19)
**2001**	2.18 (2.03-2.33)	1.88 (1.75-2.01)	2.18 (2.07-2.30)	3.11 (2.94-3.29)
**2002**	2.06 (1.91-2.20)	1.84 (1.72-1.97)	2.26 (2.14-2.37)	2.92 (2.75-3.08)
**2003**	2.01 (1.86-2.15)	1.86 (1.73-1.99)	2.13 (2.02-2.24)	2.79 (2.62-2.95)
**2004**	1.93 (1.79-2.07)	1.66 (1.54-1.78)	2.08 (1.97-2.18)	2.59 (2.43-2.74)
**2005**	1.82 (1.69-1.95)	1.64 (1.52-1.75)	2.01 (1.91-2.11)	2.52 (2.37-2.67)
**2006**	1.74 (1.61-1.87)	1.57 (1.46-1.69)	1.83 (1.73-1.93)	2.30 (2.16-2.45)
**2007**	1.61 (1.48-1.73)	1.57 (1.46-1.68)	1.83 (1.73-1.93)	2.36 (2.22-2.50)
**2008**	1.71 (1.58-1.84)	1.46 (1.35-1.57)	1.77 (1.67-1.86)	2.31 (2.17-2.45)
**2009**	1.66 (1.53-1.78)	1.48 (1.37-1.59)	1.74 (1.65-1.83)	2.43 (2.28-2.57)

**2010**	1.61 (1.49-1.73)	1.64 (1.52-1.75)	1.93 (1.83-2.03)	2.29 (2.16-2.43)
**2011**	1.63 (1.51-1.76)	1.57 (1.45-1.68)	1.84 (1.75-1.93)	2.31 (2.17-2.44)
**2012**	1.70 (1.58-1.83)	1.47 (1.36-1.57)	1.82 (1.73-1.91)	2.27 (2.14-2.40)
**2013**	1.64 (1.52-1.76)	1.54 (1.42-1.65)	1.87 (1.78-1.97)	2.30 (2.17-2.43)
**2014**	1.69 (1.57-1.82)	1.53 (1.42-1.64)	1.91 (1.81-2.00)	2.45 (2.31-2.58)
**2015**	1.62 (1.50-1.74)	1.66 (1.55-1.77)	1.94 (1.85-2.03)	2.49 (2.36-2.62)
**2016**	1.55 (1.44-1.67)	1.61 (1.50-1.72)	1.87 (1.78-1.97)	2.46 (2.32-2.59)
**2017**	1.43 (1.32-1.54)	1.61 (1.50-1.72)	1.82 (1.73-1.91)	2.32 (2.20-2.45)
**2018**	1.58 (1.46-1.70)	1.63 (1.52-1.74)	1.92 (1.83-2.01)	2.32 (2.19-2.45)
**2019**	1.48 (1.37-1.60)	1.68 (1.57-1.79)	1.88 (1.79-1.97)	2.56 (2.43-2.70)
**2020**	1.66 (1.53-1.78)	2.14 (2.01-2.27)	2.19 (2.09-2.28)	3.12 (2.97-3.27)
**2021**	1.83 (1.70-1.96)	2.25 (2.12-2.38)	2.33 (2.23-2.42)	3.44 (3.29-3.60)
**2022**	1.71 (1.58-1.83)	2.27 (2.14-2.40)	2.06 (1.97-2.15)	3.37 (3.21-3.52)
**2023**	1.55 (1.44-1.67)	2.16 (2.03-2.29)	2.10 (2.01-2.19)	3.01 (2.87-3.16)
**2024**	1.53 (1.41-1.64)	2.00 (1.88-2.12)	1.92 (1.83-2.01)	2.88 (2.74-3.02)

Supplemental Table S4. HRS-related AAMR per 100,000 stratified by Urban-Rural classification in the United States from 1999 to 2020.

	**Age Adjusted Mortality Rate (95% CI)**	
**Year**	**Metropolitan**	**Non-metropolitan**
**1999**	2.38 (2.30-2.46)	2.60 (2.42-2.78)
**2000**	2.38 (2.30-2.46)	2.53 (2.35-2.70)
**2001**	2.34 (2.26-2.41)	2.28 (2.11-2.44)
**2002**	2.22 (2.15-2.30)	2.59 (2.41-2.76)
**2003**	2.08 (2.01-2.15)	2.65 (2.47-2.83)
**2004**	2.01 (1.94-2.08)	2.39 (2.22-2.56)
**2005**	1.95 (1.88-2.01)	2.42 (2.25-2.59)
**2006**	1.78 (1.72-1.85)	2.14 (1.98-2.29)

**2007**	1.76 (1.70-1.82)	2.19 (2.03-2.35)
**2008**	1.76 (1.70-1.82)	2.14 (1.98-2.29)
**2009**	1.76 (1.70-1.82)	2.16 (2.01-2.32)
**2010**	1.81 (1.75-1.87)	2.31 (2.15-2.48)
**2011**	1.74 (1.68-1.80)	2.38 (2.21-2.54)
**2012**	1.73 (1.67-1.79)	2.33 (2.17-2.49)
**2013**	1.78 (1.72-1.84)	2.25 (2.09-2.40)
**2014**	1.82 (1.76-1.88)	2.40 (2.24-2.56)
**2015**	1.79 (1.74-1.85)	2.74 (2.56-2.92)
**2016**	1.83 (1.77-1.89)	2.29 (2.13-2.45)
**2017**	1.73 (1.67-1.79)	2.34 (2.18-2.50)
**2018**	1.78 (1.72-1.84)	2.55 (2.38-2.73)
**2019**	1.81 (1.75-1.86)	2.63 (2.46-2.81)
**2020**	2.13 (2.07-2.20)	3.18 (2.99-3.37)

Supplemental Table S5. HRS-related AAMR per 100,000 stratified by State in the United States from 1999 to 2020.

State	Age-Adjusted Rate (95% CI)
**Alabama**	1.87 (1.77-1.97)
**Alaska**	3.75 (3.34-4.16)
**Arizona**	2.20 (2.10-2.29)
**Arkansas**	1.57 (1.45-1.69)
**California**	2.57 (2.53-2.61)
**Colorado**	2.52 (2.41-2.64)
**Connecticut**	1.87 (1.76-1.99)
**Delaware**	1.79 (1.57-2.01)
**District of Columbia**	1.66 (1.39-1.93)
**Florida**	1.85 (1.81-1.90)

**Georgia**	1.43 (1.37-1.50)
**Hawaii**	2.01 (1.82-2.19)
**Idaho**	3.33 (3.09-3.57)
**Illinois**	1.44 (1.38-1.49)
**Indiana**	2.01 (1.92-2.10)
**Iowa**	1.39 (1.28-1.49)
**Kansas**	1.78 (1.65-1.91)
**Kentucky**	2.25 (2.14-2.36)
**Louisiana**	1.69 (1.60-1.79)
**Maine**	2.34 (2.14-2.53)
**Maryland**	1.26 (1.19-1.34)
**Massachusetts**	1.79 (1.71-1.87)
**Michigan**	1.72 (1.65-1.79)
**Minnesota**	1.52 (1.43-1.60)
**Mississippi**	1.80 (1.68-1.93)
**Missouri**	1.64 (1.56-1.72)
**Montana**	3.53 (3.23-3.83)
**Nebraska**	1.98 (1.81-2.14)
**Nevada**	2.18 (2.03-2.32)
**New Hampshire**	2.32 (2.12-2.53)
**New Jersey**	1.81 (1.74-1.88)
**New Mexico**	4.40 (4.16-4.65)
**New York**	1.57 (1.53-1.62)
**North Carolina**	1.83 (1.76-1.90)
**North Dakota**	3.04 (2.70-3.38)
**Ohio**	1.60 (1.55-1.66)
**Oklahoma**	2.20 (2.08-2.32)
**Oregon**	2.49 (2.36-2.61)

**Pennsylvania**	1.73 (1.67-1.79)
**Rhode Island**	2.13 (1.91-2.35)
**South Carolina**	2.46 (2.35-2.58)
**South Dakota**	3.22 (2.89-3.55)
**Tennessee**	2.20 (2.10-2.29)
**Texas**	2.54 (2.49-2.60)
**Utah**	1.66 (1.52-1.81)
**Vermont**	2.48 (2.18-2.78)
**Virginia**	1.66 (1.59-1.74)
**Washington**	2.26 (2.17-2.35)
**West Virginia**	2.74 (2.56-2.93)
**Wisconsin**	1.82 (1.73-1.90)
**Wyoming**	3.68 (3.26-4.11)

Supplemental Table S6. HRS-related CMR stratified by age groups in the United States from 1999 to 2024.

	**Crude Mortality Rate (95% CI)**		
**Year**	**Young Adults (25-44)**	**Middle-Aged Adults (45-64)**	**Old Adults (65-85+)**
**1999**	0.68 (0.62-0.73)	3.19 (3.05-3.34)	5.22 (4.98-5.46)
**2000**	0.66 (0.60-0.71)	3.21 (3.07-3.35)	5.01 (4.77-5.24)
**2001**	0.65 (0.59-0.70)	3.06 (2.93-3.20)	4.92 (4.69-5.15)
**2002**	0.63 (0.58-0.68)	3.14 (3.01-3.27)	4.64 (4.42-4.86)
**2003**	0.58 (0.53-0.63)	2.98 (2.85-3.11)	4.58 (4.35-4.80)
**2004**	0.56 (0.51-0.61)	2.98 (2.85-3.11)	4.05 (3.84-4.26)
**2005**	0.49 (0.45-0.54)	2.86 (2.74-2.98)	4.10 (3.89-4.30)
**2006**	0.46 (0.42-0.51)	2.69 (2.57-2.81)	3.72 (3.53-3.92)
**2007**	0.46 (0.41-0.51)	2.68 (2.57-2.80)	3.65 (3.46-3.85)

**2008**	0.47 (0.42-0.52)	2.74 (2.62-2.86)	3.41 (3.23-3.60)
**2009**	0.46 (0.42-0.51)	2.78 (2.66-2.89)	3.37 (3.19-3.55)
**2010**	0.52 (0.47-0.57)	2.86 (2.75-2.98)	3.44 (3.26-3.62)
**2011**	0.48 (0.43-0.53)	2.82 (2.70-2.93)	3.39 (3.21-3.56)
**2012**	0.40 (0.36-0.45)	2.91 (2.80-3.03)	3.38 (3.21-3.56)
**2013**	0.47 (0.43-0.52)	2.85 (2.74-2.97)	3.42 (3.25-3.59)
**2014**	0.42 (0.37-0.46)	2.90 (2.79-3.02)	3.82 (3.64-3.99)
**2015**	0.51 (0.47-0.56)	3.02 (2.90-3.14)	3.55 (3.38-3.72)
**2016**	0.52 (0.48-0.57)	2.80 (2.68-2.91)	3.68 (3.51-3.84)
**2017**	0.44 (0.40-0.49)	2.75 (2.64-2.86)	3.66 (3.49-3.82)
**2018**	0.56 (0.51-0.61)	2.66 (2.55-2.77)	3.83 (3.66-4.00)
**2019**	0.58 (0.53-0.63)	2.79 (2.68-2.90)	3.79 (3.62-3.95)
**2020**	0.87 (0.80-0.93)	3.33 (3.21-3.46)	4.08 (3.91-4.24)
**2021**	0.95 (0.88-1.01)	3.63 (3.50-3.76)	4.23 (4.06-4.40)
**2022**	0.91 (0.85-0.97)	3.28 (3.15-3.40)	4.32 (4.15-4.49)
**2023**	0.82 (0.76-0.88)	3.06 (2.94-3.17)	4.31 (4.14-4.47)
**2024**	0.81 (0.75-0.87)	2.74 (2.63-2.86)	4.19 (4.03-4.36)

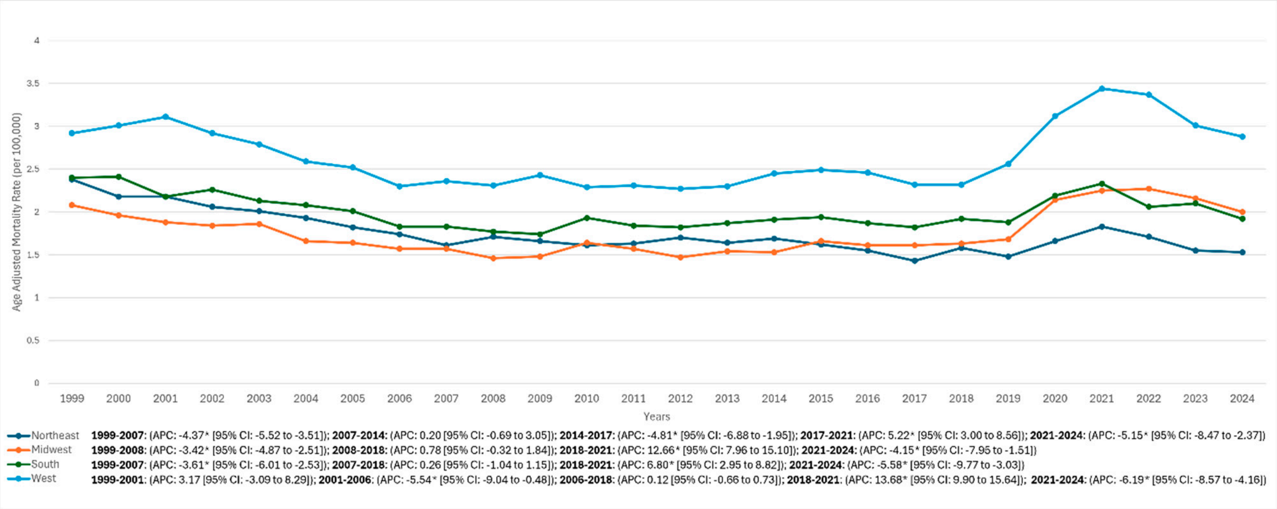

Supplementary Figure S1. HRS-associated AAMRs per 100,000 stratified by census region in the United States from 1999 to 2024.

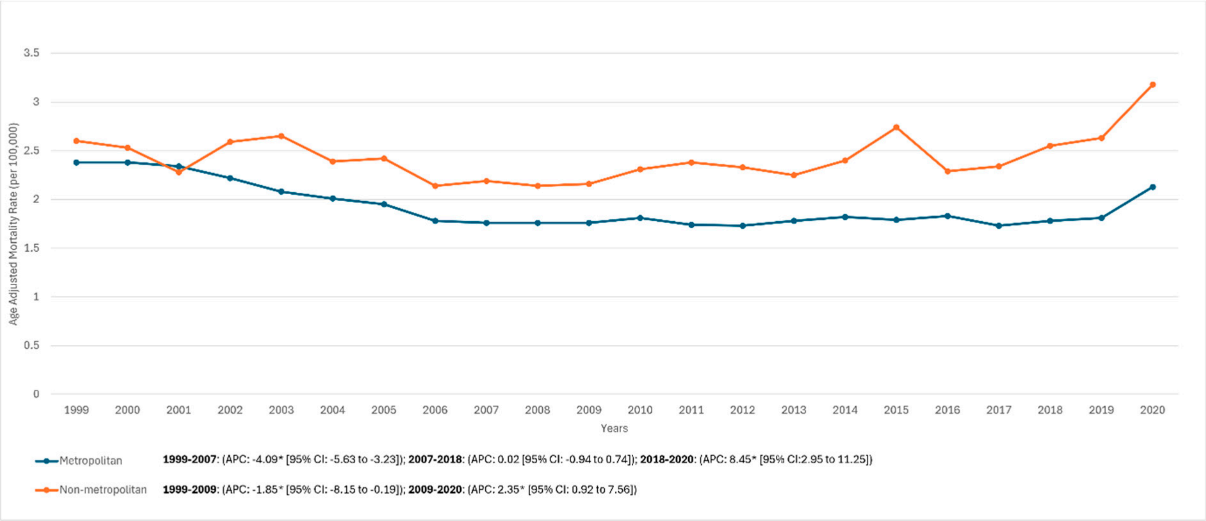

Supplementary Figure S2. HRS-associated AAMRs per 100,000 stratified by urbanization in the United States from 1999 to 2020.

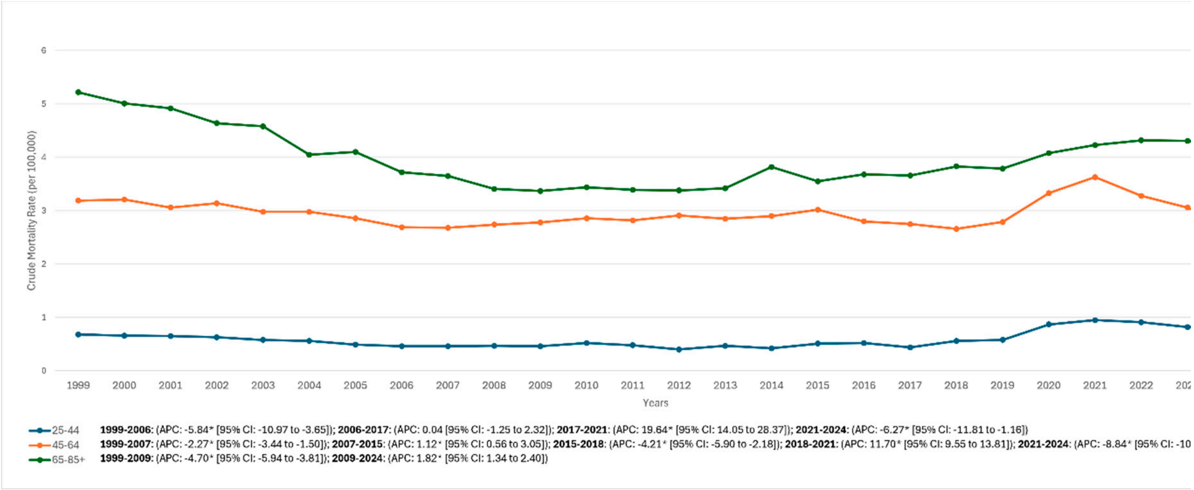

Supplementary Figure S3. HRS-associated AAMRs per 100,000 stratified by age groups in the United States from 1999 to 2024.
